# Epidemiology of Using Electronic Cigarettes in Saudi Arabia: A Systematic Review

**DOI:** 10.7759/cureus.79870

**Published:** 2025-03-01

**Authors:** Talal Alaboodi, Thamer A Al Sayari, Mostafa Kofi, Mansour K Almadi, Mohammed S Sabr

**Affiliations:** 1 Family and Community Medicine, Prince Sultan Military Medical City, Riyadh, SAU

**Keywords:** associated factors, electronic cigarettes, motives, prevalence, systematic review

## Abstract

The objective of this study is to assess the prevalence and factors influencing e-cigarette use in Saudi Arabia. We did a comprehensive search of multiple academic databases, including PubMed, SCOPUS, Science Direct, Web of Science, and Cochrane Library. Eligible studies published between January 2021 and September 2024 were screened, and the Joanna Briggs Institute (JBI) tool was used to assess the risk of bias. The study analyzed data from 15 trials involving 6,736 participants, of whom 3,872 (57.5%) were male. The prevalence of e-cigarette use varied between 7.2% among university students and 79.8% among young adults (18 to 30 years), with an overall median prevalence of 36.6%. The study found that factors such as peer influence, a variety of available flavors, male gender, curiosity, independent living situations, and perceptions of e-cigarettes as a safer and more economical alternative to traditional cigarettes might have contributed to increased usage among consumers. The findings highlight the urgent need for public health initiatives to address e-cigarette use, particularly among adolescents and young adults, to mitigate its impact on public health. Finally, we hope this study provides valuable insights for policymakers and healthcare professionals in Saudi Arabia.

## Introduction and background

Worldwide e-cigarette use is growing in popularity [1,2]. These are devices that create an aerosol that is breathed in by heating an e-liquid solution using an electrically powered coil [3]. Many of the modern variants mimic computer gadgets, pens, and other goods, even though the original devices looked like cigarettes [4].

The E-liquids typically, but not always, contain nicotine. Flavors and additives are dissolved in a solution of propylene glycol or glycerin, which is the primary carrier utilized. Therefore, in addition to e-cigars and e-pipes, there are two more forms of e-cigarettes in use, namely the electronic nicotine delivery systems (ENDS) and occasionally the electronic non-nicotine delivery systems (ENNDS) [5]. Nevertheless, secondhand exposure to aerosols by non-users or users is hazardous due to the presence of nicotine and other hazardous substances that are frequently present in e-cigarette emissions.

Electronic cigarettes, also known as e-cigarettes, are defined by the FDA as battery-operated devices that can deliver nicotine and other chemicals [6]. Even though e-cigarettes are known to be dangerous and harmful to the respiratory system, they are frequently mistakenly believed to be less harmful than tobacco products [7]. Nonetheless, they are extensively marketed and utilized globally, especially among young adults, especially those from affluent backgrounds [8]. Regrettably, the media's portrayal of e-smoking as comparatively less dangerous than traditional smoking has led to its rising popularity [9]. Although there is a wealth of information regarding the dangers of both tobacco use and e-cigarettes, little is known about how common e-cigarettes are in Saudi Arabia and what is driving their rising popularity there [10].

The growing prevalence of e-cigarettes, especially among adolescents and young people, prompts worry about the normalization of smoking habits and the risk of nicotine addiction [3]. There isn't much research on e-cigarette use in Saudi Arabia, even though the product is gaining popularity throughout the world. To create public health interventions that are successful and customized to the social and cultural context of the area, it is imperative to comprehend the prevalence and associated factors.

By methodically examining studies that look into e-cigarette use in Saudi Arabia, this review hopes to fill in the information by providing guidance for future research and informing health policy. This systematic review's main goals are to determine the prevalence and factors linked to e-cigarette usage and evaluate how common e-cigarette use is in Saudi Arabia. The review specifically looks at social effects, behavioral patterns, and demographic traits related to e-cigarette use.

## Review

Methods

This systematic review follows the Preferred Reporting Items for Systematic Reviews and Meta-Analyses (PRISMA) guidelines [11]. A structured search was performed using PubMed, Web of Science, SCOPUS, Science Direct, and Cochrane Library to identify English-language studies examining e-cigarette prevalence and associated factors in Saudi Arabia. The search incorporated relevant keywords, and multiple reviewers who independently assessed the results of their search for relevance and quality. Meta-analysis was not done due to heterogeneity among the included studies. We conducted a narrative synthesis instead.

Eligibility Criteria

The inclusion criteria for this study were as follows: studies that provided data on the prevalence of e-cigarette usage and its associated factors, research conducted within Saudi Arabia, peer-reviewed publications, studies published between January 2021 and September 2024, and studies available in the English language. The exclusion criteria were studies lacking quantitative data on e-cigarette prevalence, research conducted outside Saudi Arabia, studies published in languages other than English, animal or laboratory-based research, and non-primary research articles such as reviews, case reports, and opinion pieces.

Data Extraction

The accuracy of search results was validated using Rayyan (Qatar Computing Research Institute, Doha, QAT) [12]. To assess the relevance of the identified studies, the inclusion and exclusion criteria were applied to both titles and abstracts. Studies that met the inclusion criteria underwent a detailed review by the research team. Any disagreements regarding inclusion were resolved through consensus. A standardized data extraction form was utilized to document essential study details, such as study titles, authors, publication year, Saudi city, participant characteristics, population type, prevalence of e-cigarette use, and key findings.

Strategy for Data Synthesis

A qualitative synthesis was conducted by summarizing key research findings and study characteristics from relevant sources. Following the completion of the data collection process for this systematic review, the most effective approach to synthesizing the extracted data was determined to ensure optimal utilization of the included studies.

Risk of Bias Assessment

The quality of the studies included in this analysis was evaluated using the Joanna Briggs Institute (JBI) [13] critical appraisal tool designed for prevalence studies. This tool comprises nine criteria, with responses categorized as positive (1), uncertain, or irrelevant, with 0 being the lowest possible score. Based on total scores, studies were classified into three quality levels: low (below 4), moderate (5-7), and high (above 8). To maintain consistency and accuracy in quality assessment, researchers conducted independent evaluations, and any discrepancies were resolved through collaborative discussion.

Results

Systematic Search Outcomes

**Figure 1:**
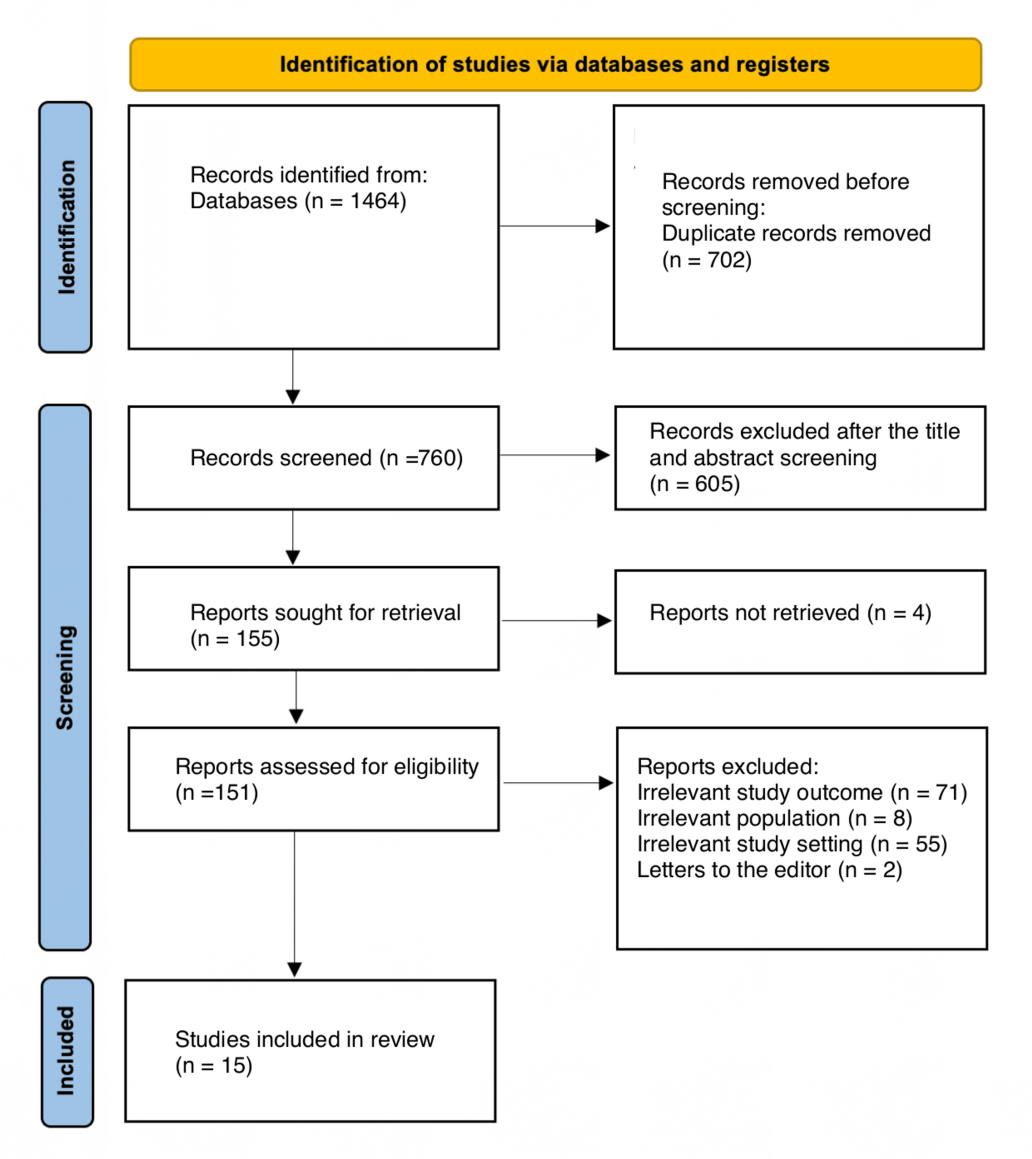
A PRISMA diagram summarizing the study decisions PRISMA: Preferred Reporting Items for Systematic Reviews and Meta-Analyses

Following the removal of 702 duplicates, a systematic search yielded 760 study papers. After 760 studies' titles and abstracts were reviewed, 605 papers were rejected. Out of the 155 reports that needed to be obtained, four articles were not found; 151 articles passed the full-text screening procedure; 71 were dismissed as the study results were irrelevant, eight because the type of population was irrelevant, two were editor's letters, and 55 were irrelevant study settings. The eligibility requirements were satisfied by 15 research publications that have been incorporated in this systematic review. Figure 1 depicts a diagram of the approach used to select the literature. 

Sociodemographics of the Comprised Participants and Studies

Table 1 displays the sociodemographic information from the research articles. Our data included 15 trials with 6,736 participants of which 3,872 (57.5%) were males. All of the included articles were cross-sectional studies [14-28]. Four studies were conducted in Riyadh [16,19,21,28], two in Al-Ahsa [18,22], three in Jeddah [24,26,27], one in Mecca [14], one in Jazan [16], one in Shaqra [17], one in Taif [20], one in Al-Madinah [23], and one in Al-Kharj [25].

**Table 1:** Sociodemographic parameters of the involved populations NM: Not mentioned

Authors	Study design	City	Participants	Mean age	Males (%)
Rayes et al., 2023 [14]	Cross-sectional	Mecca	534	16.8 ± 0.9	337 (44.4%)
Aqeeli et al., 2024 [15]	Cross-sectional	Jazan	1,187	21	703 (59.2%)
Khanagar et al., 2024 [16]	Cross-sectional	Riyadh	164	22.8 ± 0.9	3 (1.8%)
Algassim et al., 2024 [17]	Cross-sectional	Shaqra	290	20.5 ± 1.9	192 (66.2%)
Alabdulqader et al., 2024 [18]	Cross-sectional	Al-Ahsa	472	15.8 ± 1.8	472 (100%)
AlHumaidan et al., 2024 [19]	Cross-sectional	Riyadh	476	21.3 ±2.4	98 (20.6%)
Albgami et al., 2023 [20]	Cross-sectional	Taif	319	≥18	319 (100%)
Khan et al., 2023 [21]	Cross-sectional	Riyadh	388	NM	311 (80%)
Al Rajeh et al., 2023 [22]	Cross-sectional	Al-Ahsa	325	24.7 ± 5.1	325 (100%)
Alzahrani et al., 2023 [23]	Cross-sectional	Al-Madinah	519	20 ± 2	245 (47.2%)
Ghamri et al., 2023 [24]	Cross-sectional	Jeddah	133	NM	81 (60.9%)
Ali, 2024 [25]	Cross-sectional	Al-Kharj	570	NM	124 (21.8%)
Alzahrani et al., 2022 [26]	Cross-sectional	Jeddah	465	>18	271 (58.3%)
Alzahrani et al., 2021 [27]	Cross-sectional	Jeddah	399	21.7 ± 2.9	178 (44.6%)
Alammari et al., 2022 [28]	Cross-sectional	Riyadh	495	18-74	213 (43%)

Clinical Outcomes

The clinical data are presented in Table 2. Half of the included studies comprised university students [15-17,20,23,24], two comprised adolescents [14,18], two comprised young adults [19,21], and two comprised the general population [22,25]. The prevalence of e-cigarette use ranged from 7.2% [15] among university students to 79.8% [19] among young adults, with a total prevalence of 2,056 (30.5%). The high prevalence in young adults is attributed to the fact that teenagers and young adults are using e-cigarettes at a much higher rate due to the devices' accessibility and sales [16-18,25]. The prevalence of e-cigarette use was associated with peer pressure [18], the presence of many flavors [19,22], male gender [15,19], curiosity [22], participants who live alone or with friends [19], belief that e-cigarettes are safer than traditional cigarettes [19,23], cost savings over traditional cigarettes [22], and lower levels of education [25].

**Table 2:** Clinical parameters and outcomes of the comprised research JBI: Joanna Briggs Institute critical assessment criteria

Authors	Population type	Prevalence of e-cigarette use (%)	Main outcomes	JBI
Rayes et al., 2023 [14]	Adolescents	109 (20.6%)	Even a small amount of smoking experience among teenage smokers is associated with favorable attitudes about smoking. Teenagers who use e-cigarettes frequently also use other combustible tobacco products.	High
Aqeeli et al., 2024 [15]	University students	85 (7.2%)	The following are important variables that impact dependence: male gender, length of e-cigarette use, daily use, and prior attempts to quit. Those who use drugs for longer periods and men are more likely to become dependent.	Moderate
Khanagar et al., 2024 [16]	University students	72 (43.9%)	Individuals with a history of e-cigarette use are more likely to start smoking traditional cigarettes. Teenagers and young adults are using e-cigarettes at a much higher rate due to the device's accessibility and sales.	Moderate
Algassim et al., 2024 [17]	University students	58 (20.1%)	The average age of e-cigarette users was 20.5 years, and the Shaqra Governorate had the greatest prevalence of e-cigarette use.	High
Alabdulqader et al., 2024 [18]	Adolescents	83 (17.6%)	A tiny percentage of participants admitted to using e-cigarettes, mostly as a result of peer pressure. Online purchases accounted for the majority of e-cigarette purchases, and the majority of e-cigarette users planned to stop using them or had already tried.	Moderate
AlHumaidan et al., 2024 [19]	Young adults	380 (79.8%)	The main justification given by young Saudi adults for using e-cigarettes was their flavors. Moreover, many thought that switching to e-cigarettes was a risk-free substitute for stopping traditional smoking. Currently, men and non-Saudis are more likely to use e-cigarettes. High consumption was seen among respondents who were single or living with friends, and thought e-cigarettes were safer than traditional cigarettes.	Moderate
Albgami et al., 2023 [20]	University students	128 (40.1%)	A sizable fraction of participants thought e-cigarettes were safer than traditional cigarettes, and many were ignorant of the possible health risks associated with using them. The study emphasizes the necessity of public health initiatives and campaigns to dispel myths about e-cigarette safety and raise public knowledge of the dangers connected to use.	Moderate
Khan et al., 2023 [21]	Young adults	234 (60%)	Higher education levels are associated with a greater level of awareness of the negative effects of e-cigarettes, which is one factor that influences the use of these devices.	Moderate
Al Rajeh et al., 2023 [22]	General population	109 (33.5%)	Present smokers stated that they use e-cigarettes for several reasons, including curiosity, flavor preference, cost savings over traditional cigarettes, and plans to cut back on or give up traditional cigarette smoking.	Moderate
Alzahrani et al., 2023 [23]	University students	123 (23.7%)	The widespread usage of e-cigarettes was facilitated by a false belief regarding their safety.	Moderate
Ghamri et al., 2023 [24]	University students	90 (69.2%)	Many medical students have found that using e-cigarettes or vapes helps them stop smoking, and it is thought of as a bridge for those who want to cut back on their smoking.	Moderate
Ali, 2024 [25]	General population	124 (21.8%)	Significant variations were also observed in educational attainment, with higher levels of education being linked to a lower use of e-cigarettes. Young individuals and those with a history of tobacco product use are the most common demographics among e-cigarette users.	Moderate
Alzahrani et al., 2022 [26]	General population	241 (51.8%)	The usage of e-cigarettes is extremely common, and its numbers are sharply rising with time in comparison to previous studies. Entertainment has been found to be the primary driver of its appeal, with helping people quit traditional smoking coming in second.	Low
Alzahrani et al., 2021 [27]	Medical students	146 (36.6%)	According to prior experience with e-cigarettes, around 25% of the medical students participating in this study were expected to be favorably inclined to use e-cigarettes medically as a smoking cessation approach or as a means of reducing risk for patients.	Moderate
Alammari et al., 2022 [28]	General population	74 (41.6%)	Since e-cigarettes are widely used, it is necessary to increase public knowledge of their negative effects. It is advised that policymakers step up health initiatives to increase public knowledge of this important health concern and to thwart commercial corporations' marketing campaigns that encourage the use of e-cigarette devices.	Moderate

Discussion

This systematic review draws attention to the growing trend of e-cigarette usage in Saudi Arabia, where prevalence rates fluctuate among several demographic segments. The prevalence of e-cigarette use ranged from 7.2% [15] among university students to 79.8% [19] among young adults, with overall median prevalence of 36.6% in Saudi Arabia. This was higher than Patil et al. who reported a 10.6% to 27.7% e-cigarette use prevalence among Saudi medical students in a similar comprehensive review [29]. Our prevalence was also higher than the global prevalence observed by Salari et al. (16.8%) [30] and Kim et al. (15.3%) [31].

This review found some factors that could be associated with the high prevalence in young adults, which is attributed to the fact that teenagers and young adults are using e-cigarettes at a much higher rate due to the device's accessibility and sales [16-18,25]. This observation aligns with earlier research showing that exposure to and positive opinions of e-cigarette advertising promoted adolescent e-cigarette use [32]. Due to the prominent online presence of e-cigarette companies and the ease with which youth may obtain information about e-cigarettes via social media, many of them are encouraged to look for vaping products without giving them any thought or validation [33,34]. Additionally, there are modest health warning signals and a loose age verification process on the internet for e-cigarette items [34]. These factors have led to a rise in the frequency of successful e-cigarette transactions by minors. At the moment, each country has different laws limiting the sale of e-cigarettes, particularly to people above a specific age. For example, the Tobacco & Vaping Products Act in Canada prohibits the sale of e-cigarettes to minors under the age of 18 [35] and controls the manufacturing, distribution, labeling, and advertising of e-cigarettes sold in the nation [36,37].

We found that several studies reported that some factors may be associated with higher e-cigarette use such as peer pressure [18], the presence of many flavors [19,22], male gender [15,19], curiosity [22], participants who live alone or with friends [19], belief that e-cigarettes were safer than traditional cigarettes [19,23], cost savings over traditional cigarettes [22], and lower levels of education [25]. In line with our results, Chapman and Wu reported [38] that even though a history of cigarette smoking was found to be a prevalent correlate of e-cigarette usage, a significant percentage of teenagers and young adults who had never smoked were acquainted with e-cigarettes. Among young adults, e-cigarette use was not always linked to attempts to give up tobacco smoking. Adults reported using e-cigarettes most frequently to replace tobacco, albeit not necessarily to stop smoking. The usage of e-cigarettes by young people (new users who had never used tobacco) and adults (previous or present tobacco users) differed slightly, according to reviewed studies [38]. Kim et al. also reported that gender disparities were noted in over half of the research, with men often exhibiting greater lifetime, current, and dual e-cigarette use rates than women. Common protective factors that we identified included the perceived cost and danger of e-cigarettes, parental surveillance, the mother's educational attainment, physical exercise, parental support, internal developmental assets, and academic accomplishment. Additionally, we found that the following were typical risk factors: friends smoking, drinking, smoking marijuana, family members smoking, using other drugs or substances, being exposed to internet commercials and advertisements, performing poorly in school, and believing that e-cigarettes are less hazardous [31].

There are multiple limitations on this systematic review. First, the methods, sample sizes, and populations of the studies that made up the review vary, which would restrict how broadly applicable the results are. Secondly, the majority of research studies depended on self-reported data, which may be influenced by social desirability or recall bias. Third, the review can be constrained by the available papers; some pertinent studies might not have been published or might have been left out because of linguistic limitations. Despite these limitations, we hope this review provides valuable insights into e-cigarette use trends in Saudi Arabia. Future research should address key gaps in the literature, including the lack of longitudinal studies, which limits understanding of usage trends over time. 

## Conclusions

The use of E-cigarettes is high in Saudi Arabia, especially among young adults and university students. Studies in this review attributed this to the device's easy access by this specific population via online sales. The results emphasize the necessity of focused public health initiatives to combat the growing trend of e-cigarette use, especially in adolescents and young adults. Policymakers and healthcare practitioners seeking to lessen the impact of e-cigarettes on public health in Saudi Arabia may find this review's identification of important determinants of e-cigarette use to be insightful. Sustained investigation and observation are necessary to track e-cigarette usage patterns and assess the success of local public health initiatives.
